# Supplementary material for: Characterization of growth and development of sorghum genotypes with differential susceptibility to Striga hermonthica
Source: J Exp Bot. 2021 Aug 19;72(22):7970–83. doi: 10.1093/jxb/erab380 (PMC8643648; doi:10.1093/jxb/erab380)
Supplement: erab380_suppl_Supplementary_Figures_S1-S5 [file erab380_suppl_supplementary_figures_s1-s5.pdf]

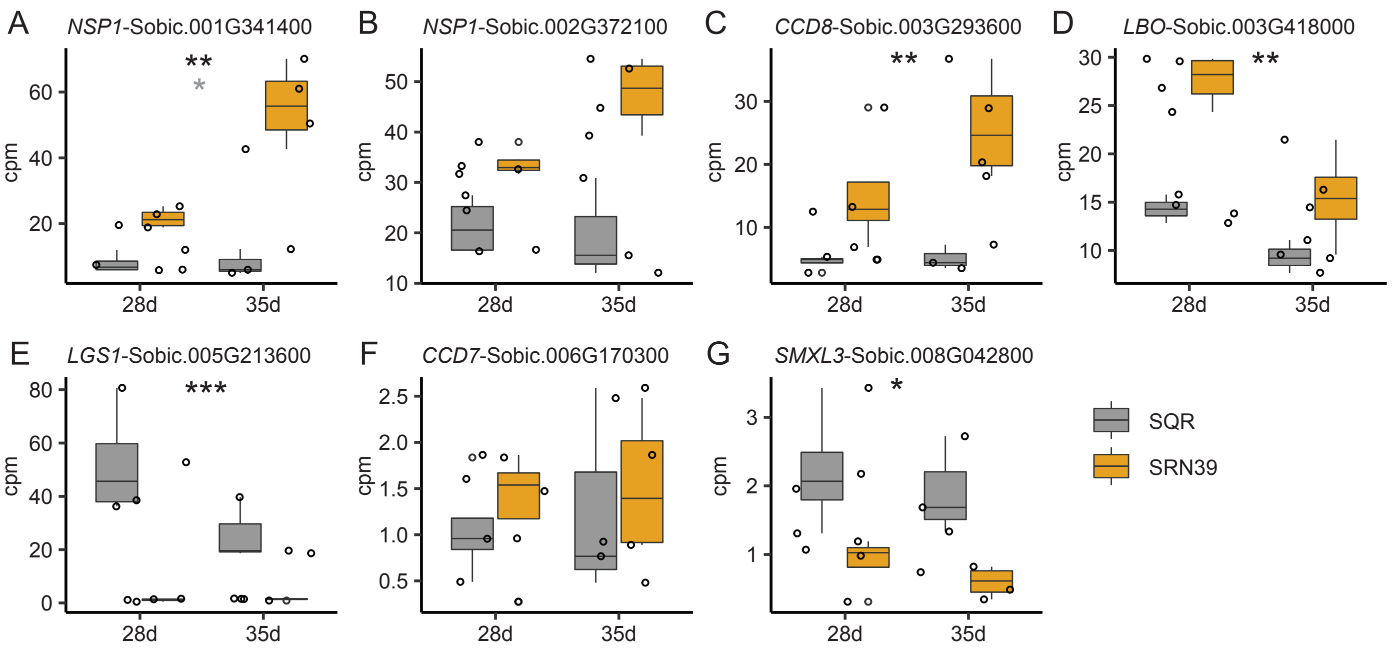

Figure S1. Expression of strigolactone biosynthesis and signaling pathway genes in roots of 28 and 35 day old plants of Shanqui Red (SQR) and SRN39. In all cases the x-axis denotes plant age (28d – 28 day old, 35d – 35 day old plants). (A-B) SRN39=orange; SQR=gray. Transcripts in A, B, D-G has been previously reported as differentially expressed between SQR and SRN39 in (Bellis et al., 2020). The boxplots denote data spanning from the 25th to the 75th percentile and are centered to the data median. Dots represent individual values. Black asterisks denote a significant adjusted p-value for term genotype, while grey asterisk indicate significant adjusted p-value for the genotype*time interaction. *** p-value < 0.001, ** p-value < 0.01, * p-value < 0.05, (n=4).

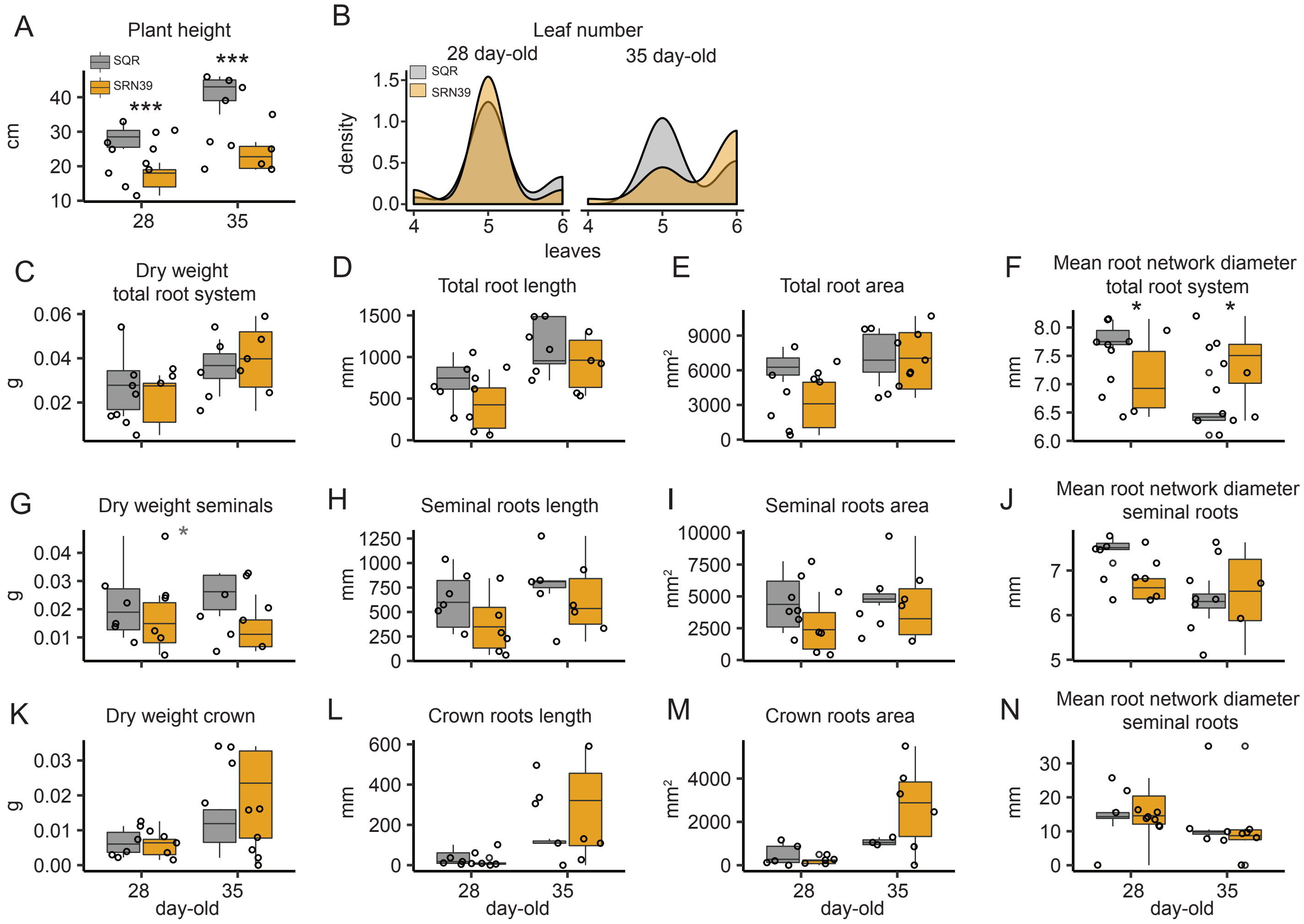

Figure S2. Phenotypic characterization of 28 and 35 day old plants of Shanqui Red (SQR) and SRN39. (A-N) SRN39=orange; SQR=gray (A) Plant height and (B) leaf number, dry weight, root length, root area and mean root network diameter of (C-F) total root system, (G-J) seminal roots and (K-N) crown roots. The boxplots denote data spanning from the 25th to the 75th percentile and are centered to the data median. Dots represent individual values. Black asterisks denote a significant p-value for a post-hoc pairwise comparison between each genotype withing a timepoint by the least square method. Gray asterisks denote a significant p-value for a genotype term according to a two-way ANOVA. *** p-value < 0.001, ** p-value < 0.01, * p-value < 0.05, (n=6 for A, C-N; n= 15-22 for B).

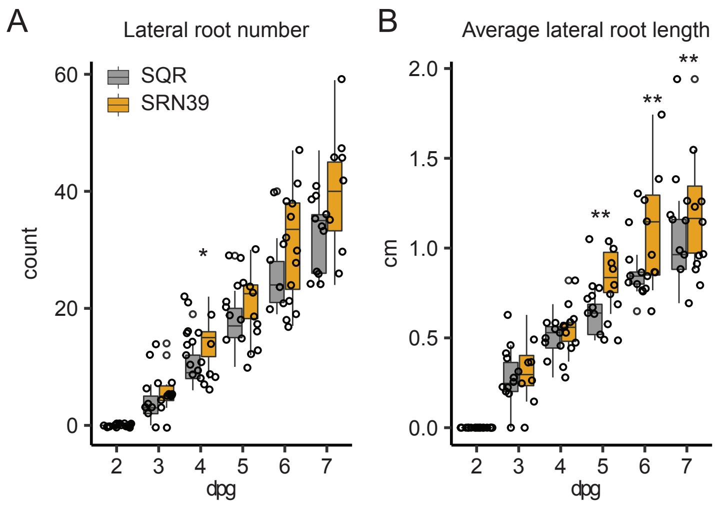

Figure S3. Root system architecture of Shanqui Red (SQR) and SRN39. In all cases the x-axis denotes days post germination (dpg). (A-B) SRN39=orange; SQR=gray. (A) Lateral root number, (B) average lateral root length. The boxplots denote data spanning from the 25th to the 75th percentile and are centered to the data median. Dots represent individual values. Asterisks denote a significant p-value for each timepoint between genotypes by the least square method. *** p-value < 0.001, ** p-value < 0.01, * p-value < 0.05, (n=10).

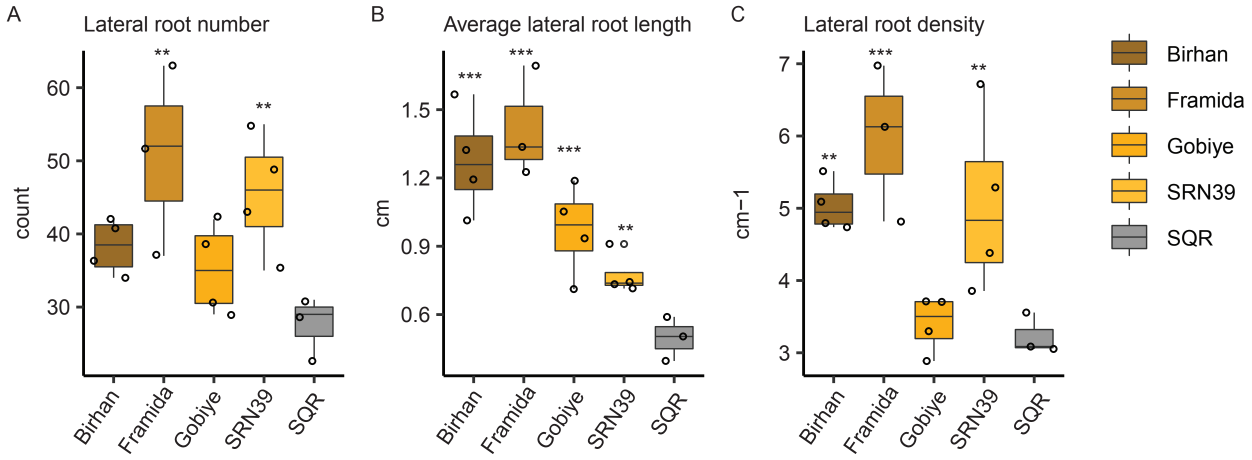

Figure S4. Root system architecture of seven day old seedlings of Shanqui Red (SQR) and sorghum varieties with the *lgs1* mutation (Birham, Framida, Gobiye and SRN39). (A) Lateral root number, (B) average lateral root number, (C) lateral root density. The boxplots denote data spanning from the 25th to the 75th percentile and are centered to the data median. Dots represent individual values. Asterisks denote a significant p-value for each timepoint between genotypes by the least square method. *** p-value < 0.001, ** p-value < 0.01, * p-value < 0.05, (n=4).

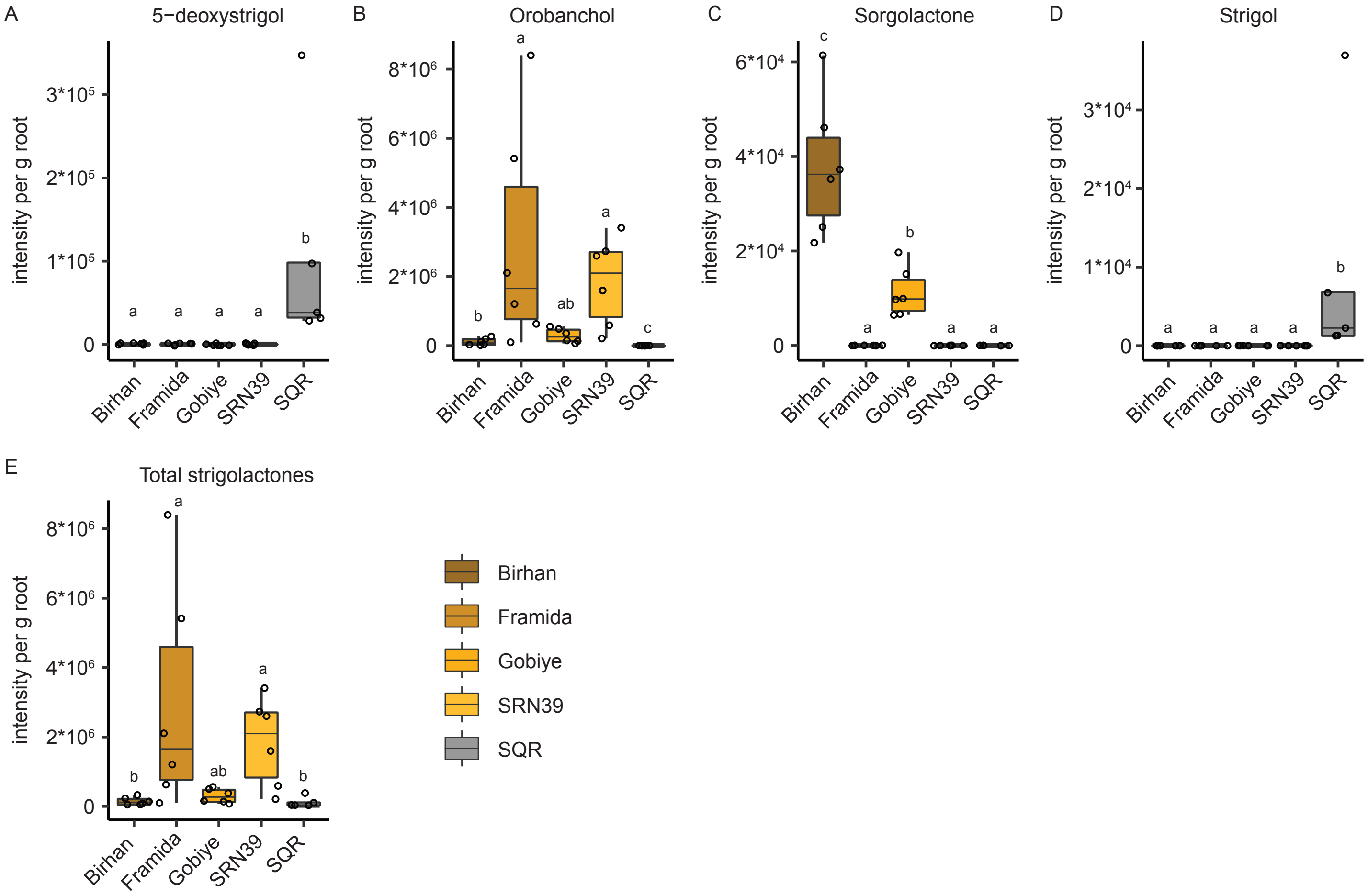

Figure S5. Strigolactone composition in root exudates of Shanqui Red (SQR) and sorghum varieties with the *lgs1* mutation (Birham, Framida, Gobiye and SRN39. Abundance (expressed as intensity per gram of fresh root weight) of (A) 5-deoxystrigol, (B) orobanchol, (C) sorgolactone, (D) strigol and (E) total strigolactones in exudates of 14 day old plants (n=6). The boxplots denote data spanning from the 25th to the 75th percentile and are centered to the data median. Dots represent individual values. Statistical analysis has been performed with one-way ANOVA with Tukey post-hoc test (adjusted p-value<0.05). Different letters indicate significance of difference between tested genotypes.
